# Octamer binding transcription factor-4 expression is associated with cervical cancer malignancy and histological differentiation: a systematic review and meta-analysis

**DOI:** 10.1042/BSR20182328

**Published:** 2019-05-10

**Authors:** Zi-ye Gao, Xiao-bo Liu, Feng-mei Yang, Ling Liu, Jin-zhang Zhao, Bo Gao, Sheng-bao Li

**Affiliations:** 1 Department of Oncology, Taihe Hospital, Hubei University of Medicine, Shiyan 442000, Hubei, China; 2 Department of Gastroenterology, Taihe Hospital, Hubei University of Medicine, Shiyan 442000, Hubei, China; 3 Department of Obstetrics and Gynecology, Taihe Hospital, Hubei University of Medicine, Shiyan 442000, Hubei, China; 4 Department of Neonatology, Northwest Women and Children Hospital, Xi’an 710000, Shanxi, China; 5 Department of Pediatric medicine, Northwest Women and Children Hospital, Xi’an 710000, Shanxi, China; 6 Department of Laboratory Medicine, Taihe Hospital, Hubei University of Medicine, Shiyan 442000, Hubei, China

**Keywords:** Cervical cancer, Meta-analysis, OCT-4, Systematic review, Tumor stem cells

## Abstract

**Objective:** In this work, the relationship between octamer binding transcription factor 4 (OCT-4) expression and the clinicopathological features of cervical cancer (CC) is evaluated in detail.

**Methods:** The library databases Pubmed, Embase, Cochrane library, Wan Fang and Chinese National Knowledge Infrastructure (CNKI) were searched for research related to these concepts published from the time the databases were established until May 2018. The obtained studies are screened, extracted, and evaluated according to the inclusion and exclusion criteria, and meta-analysis is carried out via RevMan 5.3.

**Results:** Ten case–control studies, including 408 cases of CC, 164 cases of cervical intraepithelial neoplasia (CIN), and 148 cases of normal cervix, are included in the analysis. Results show that OCT-4 levels are statistically significantly different between the CC and normal cervical tissue groups (odds ratio (OR) = 15.59, 95% confidence interval (CI): 8.70, 27.94), the CC and CIN groups (OR = 5.64, 95% CI: 3.23, 9.86), the CIN and normal cervical tissues groups (OR = 7.13, 95% CI: 2.41, 21.05), and the CC well/moderately differentiated and poorly differentiated groups (OR = 0.44, 95% CI: 0.24, 0.81). OCT-4 is not statistically significantly different between CIN I + II and CIN III tissues (OR = 0.40, 95% CI: −0.02, 0.81), the CC lymphatic and non-lymphatic metastasis groups (OR = 1.93, 95% CI: 0.83, 4.47), the FIGO I and FIGO II groups (OR = 0.79, 95% CI: 0.29, 2.13), and the adenocarcinoma and squamous cell carcinoma groups (OR = 1.55, 95% CI: 0.70, 3.44).

**Conclusions:** The available evidence suggests that OCT-4 expression is associated with CC malignancy and histological differentiation. This finding, however, is subject to quantitative studies and quality tests.

## Introduction

Cervical cancer (CC) ranks first in women’s reproductive system malignancies among developing countries, accounting for more than 50% [1]. While mortality rates have shown a downward trend since the implementation of the anti-cancer census, tumor recurrence, metastasis, and treatment non-compliance continue to adversely seriously impact patient prognosis [2]. Exploration of the pathogenesis of CC is of great significance finding new drug targets. Some cells have growth patterns and biological characteristics similar to the basic characteristics of stem cells in tumors. Cancer stem cells (CSCs), for example, feature self-renewal, proliferation, differentiation potential and other abilities [3]. In addition, CSCs play a key role in the occurrence, growth, recurrence and metastasis of CC [4,5]. Therefore, identification of CSC colonies and expression markers may be helpful in predicting the progress of CC and finding new therapeutic targets [6].

At the developmental stage, the inner cell mass of embryonic blastocysts can produce pluripotent stem cells called embryonic stem cells (ESCs). ESCs have unrestricted replication ability and can be divided into functional cell types [7]. Great progress has been made on research into the transcriptional signaling pathways of ESCs, including octamer binding transcription factor 4 (OCT-4), sexcribed regions Y-box 2 (Sox 2), Kruppel-like factor 4 (Klf4) and so on. These factors are involved in epithelial stem cell differentiation and tumor invasion [6,8].

OCT-4, also known as OCT-3 or OCT3/4, POU5F1, is a member of the POU transcription factor family (Pit-Oct-Unc (POU)-domain transcription factor family) [9]. As a universal stem cell marker, it is an important regulator in maintaining ESCs and self-renewal. OCT-4 participates in multi-directional differentiation regulation in embryonic development and maintains adult stem cell pluripotency [10]. The transcription factor is expressed not only in embryonic and germ cell tumors [11], but also, to a certain extent, in non-reproductive system tumor cells and tissues, such as lung cancer [12], prostate cancer [13], breast cancer [14] and so on.

At present, studies on the relationship between OCT-4 expression and CC or its clinicopathological features are limited to case studies with small samples, and the conclusions obtained are often diverse. Whether OCT-4 can be used as a surface marker of CC stem cells as a new direction for stem cell-targeted therapy is a popular research topic. In the present study, the literature at home and abroad was collected, and the correlation between OCT-4 expression level and CC was evaluated by meta-analysis to provide evidence-based reference for CC treatment.

## Materials and methods

### Literature search strategy

We followed Preferred Reporting Items for Systematic reviews and Meta-Analyses (PRISMA) guidelines [15] to conduct this systematic review. The selected databases, including Pubmed, Embase, Cochrane library, Wan Fang and Chinese National Knowledge Infrastructure (CNKI), were searched to identify suitable research regarding OCT-4 expression and the clinicopathological features of CC in case–control studies published from when the database was established up to May 2018. The search strategy included the terms Octamer binding transcription factor 4, OCT-4, OCT-3, OCT-3/4, and CC, cervical carcinoma, cervical intraepithelial neoplasia (CIN). The corresponding Chinese characters were used in Chinese databases, and the search languages included English and Chinese. Wherever possible, we traced references that had been incorporated into the literature and manually obtained the relevant conference proceedings to identify potential information that had not already been retrieved. Unpublished literature was not retrieved.

### Inclusion and exclusion criteria

Studies were included in the meta-analysis if they met the following conditions: evaluated the correlation between OCT-4 expression and clinicopathological features of CC, all cases had complete clinical and pathological data before drawing without radiotherapy or chemotherapy, immunohistochemistry (IHC) methods were used to determine OCT-4 expression, and the results were expressed as cell or intensity scores. Studies were excluded if the OCT-4 detection method for IHC positive criteria were inconsistent. Studies on animals, human xenografts and the CC cell line were also excluded. Studies were excluded if they were review, summary, systematic evaluation, the reader letter and so on.

### Data extraction

The following information was extracted from the literature: the title, first author names, the year of publication, country, the general situation of the included cases; the detection method of OCT-4; IHC methodology (primary antibody source), the clinical features of CC: lymph node metastasis, pathological grade, histological differentiation, tissue type and clinical stage. Whether all the literature was included was determined by two reviewers. They calculated the score according to the principle independently. All documents were included in the decision by two reviewers. If any differences arise, they may be decided by discussion, or consultation with the third reviewer.

### Quality assessment

The quality of the methodology of each included observational study was assessed using the Newcastle–Ottawa Quality Assessment Scale (NOS) [12].The NOS includes three aspects for cohort studies: selection, comparability and exposure or outcome. The full score was nine stars, and the high-quality study was considered as a study whose score was greater than or equal to six [16–18].

### Statistical analysis

The meta-analysis was performed by Review Manager Version 5.3 software (Cochrane Collaboration, Software Update, Oxford, United Kingdom). The association between OCT-4 expression and the clinical parameters of CC were quantitatively determined by calculating the pooled odds ratio (OR) and its 95% confidence interval (CI).

Cochran’s Q test and the *I^2^* statistic was performed to assess the heterogeneity among studies with a significance level of α = 0.1. A fixed-effect model used to calculate parameters in meta-analysis, when *P*>0.1and *I^2^* < 50%, whereas a random-effect model should used when *P*<0.1 and *I^2^* > 50%. *P*<0.1 and *I^2^*< 50%, however, within the acceptable range, a fixed-effect model should used to calculate parameters in meta-analysis. If *P*>0.1 and *I^2^* > 50%, the random-effect model can be used [19]. Publication bias was quantitatively assessed by using Egger’s test, and these operations were completed by STATA software (Stata Corporation, version 12.0,College Station, TX, U.S.A.). All *P*-values were two-tailed, *P*<0.05 was considered statistically significant publication bias.

## Results

### Literature search results and study characteristics

**Figure 1:**
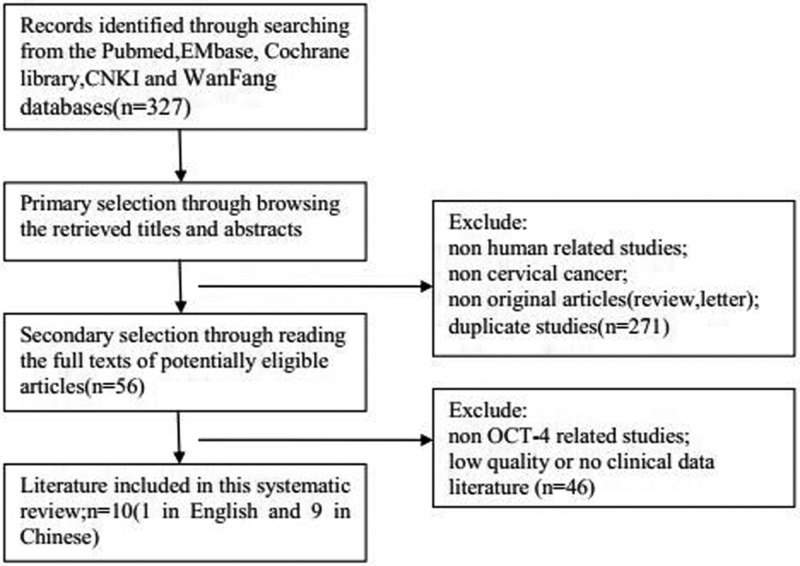
Flow chart of literature search and selection schema

A total of 327 articles were obtained from the preliminary examination. According to the above inclusion criteria and exclusion criteria, the frequencies of OCT-4 protein expression in CC were evaluated in ten case–control studies (Figure 1).

Ten studies [20–29] included 408 CC cases, nine studies [20–26,28,29] reported the expression of OCT-4 in CC and normal cervical tissues, four studies [23,24,26,27] reported OCT-4 expression in the CC tissue and CIN tissue groups, three studies [23,24,26] reported OCT-4 expression in the CIN tissue and normal cervical tissues, three studies [24,26,27] reported OCT-4 expression in the CIN Ⅰ + Ⅱ tissue and CIN Ⅲ tissues, six studies [21,22,24,26,28,29] reported the expression of OCT-4 in CC with different degrees of differentiation. Three studies [21,27,29] reported the expression of OCT-4 in different lymph node metastases, and three studies [21,25,29] reported the expression of OCT-4 in different stages of CC, eight studies [20,22–28] reported the expression of OCT-4 in CC with different pathological grades. The basic characteristics and quality of each study are shown in Table 1 and 2.

**Table 1:** Basic characteristics of included studies

First author	Year	Time to collect cases (year)	Country	Antibodies	NCM	CIN	CC tissue		Evaluation index	NOS	Reference
							SCC	AC			
Cao	2010	2006–2007	China	Santa Cruz	12	NA	22	8	a	7	[20]
Zhao	2011	2005–2010	China	Santa Cruz	10	20	30	NA	a, b, c	7	[21]
Deng	2012	2009–2010	China	Bioss Antibodies	9	NA	NA	46	a, b	7	[22]
Wan	2012	2010–2011	China	Epitomics	10	30	35	5	a, b	7	[23]
Huang	2013	2011–2012	China	Dongsheng Biotech Co. Ltd	20	30	14	6	a, b	7	[24]
Zhang	2013	2008–2012	China	Cell Signaling	28	NA	10	42	a, b	7	[25]
Tang	2013	2010–2012	China	Santa Cruz	10	30	16	4	a, b	7	[26]
Yan	2015	2005–2014	China	Fuzhou Maixin Biotech. Co. Ltd	NA	55	NA	66	b, c	7	[27]
Liu	2015	2008–2010	China	Abcam	20	NA	42	10	a, b	7	[28]
Jing	2014	2011–2012	China	Santa Cruz	28	NA	NA	43	a, b, c	8	[29]

a, The expression of OCT-4 in normal cervical tissues and CC tissues. b, OCT-4 expression in cervical tissues with different degrees of differentiation. c, OCT-4 expression in CC of different lymphatic metastases.

Abbreviations: AC, adenocarcinoma; NA, not available; NCE, normal cervical tissue; SCC, squamous cell carcinoma.

**Table 2:** The publication bias of OCT-4 expression in CC

Clinicopathological features	t	95% CI	*P*
Well/moderately vs. poorly differentiated	1.47	−7.509–8.524	0.280
Lymphatic vs. non-lymphatic metastasis	−0.56	−53.025–48.559	0.676
FIGO I vs. FIGO II	0.62	−25.033–27.614	0.645
SCC vs. AC	3.33	0.580–6.354	0.029

Abbreviations: AC, adenocarcinoma; SCC, squamous cell carcinoma.

### Results of the meta-analysis

#### Differences in OCT-4 protein expression: CC tissues vs. normal cervical tissues

**Figure 2:**
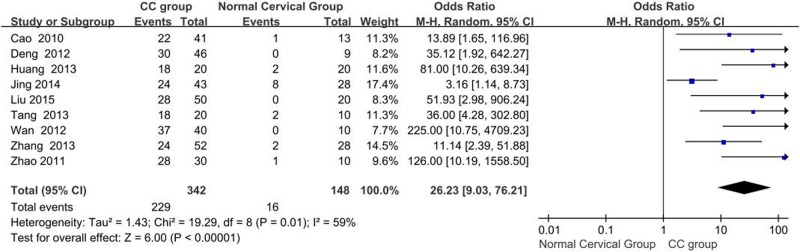
Meta analysis of the expression of OCT-4 protein in CC tissues vs. normal cervical tissues

A total of nine studies reporting OCT-4 expression in CC tissues and normal cervical tissues were obtained. *I^2^* estimates showed heterogeneity (*I^2^* = 59%, χ^2^ = 19.29, *P*=0.01) among the studies, and the random-effects model was used in the meta-analysis. The results showed that expression of OCT-4 protein in CC is significantly higher than that in normal cervical tissues (OR = 26.23, 95% CI: 9.03–76.21, *P*<0.00001, Figure 2).

#### Differences in OCT-4 protein expression : CC tissue vs. CIN tissue

**Figure 3:**
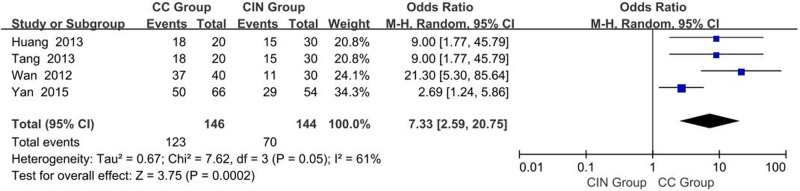
Meta analysis of the expression of OCT-4 protein in CC tissues vs. CIN tissues

A total of four studies reporting OCT-4 expression in CC tissue and CIN tissue groups were obtained. *I^2^* estimates showed heterogeneity (*I^2^* = 61%, χ^2^ = 7.62, *P*=0.05) among the studies, and the random-effects model was used in the meta-analysis. The results showed that expression of OCT-4 protein in CC tissues is significantly higher than that in CIN tissues (OR = 7.33, 95% CI: 2.59–20.75, *P*=0.0002, Figure 3).

#### Differences in OCT-4 protein expression : CIN tissue vs. normal cervical tissues

**Figure 4:**
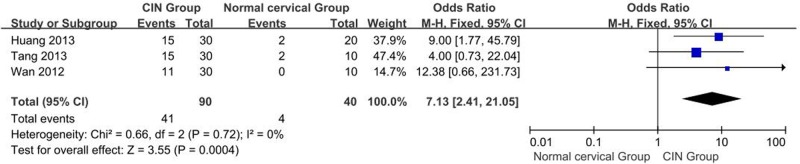
Meta analysis of the expression of OCT-4 protein in normal cervical tissues vs. CIN tissues

A total of three studies reporting OCT-4 expression in normal cervical tissues and CIN tissues were obtained. *I^2^* estimate indicated has no heterogeneity (*I^2^* = 0%, χ^2^ = 0.66, *P*=0.72) among the studies, and the fixed-effects model used in this meta-analysis. The results showed that expression of OCT-4 protein in CIN tissues is significantly higher than that in normal cervical tissues (OR = 7.13, 95% CI: 2.41–21.05, *P*=0.0004, Figure 4).

#### Differences in OCT-4 protein expression: CIN Ⅰ + Ⅱ tissue vs. CIN Ⅲ tissue

**Figure 5:**
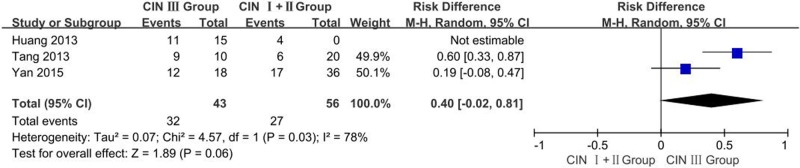
Meta analysis of the expression of OCT-4 protein in CIN Ⅰ + Ⅱ tissues vs. CIN Ⅲ tissues

A total of three studies reported OCT-4 expression in the CIN Ⅰ + Ⅱ tissue and CIN Ⅲ tissue groups. The *I^2^*estimate indicated has heterogeneity (*I^2^* = 78%, χ^2^ = 4.57, *P*=0.03) among the studies, and the random-effects model used in this meta-analysis. The results showed that the expression of OCT-4 protein in CIN Ⅲ tissues was higher than that in CIN Ⅰ + Ⅱ tissues, however, the difference was not significantly (OR = 0.40, 95% CI: −0.02–0.81, *P*=0.06, Figure 5).

#### Differential expression of OCT-4 in CC

##### Differential expression of OCT-4 in CC: poorly differentiated vs. well and moderately differentiated

**Figure 6:**
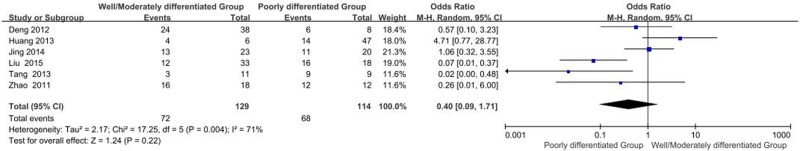
Meta analysis of expression of OCT-4 protein in CC tissues with different degrees of differentiation

A total of six studies reporting OCT-4 expression in the CC with different degrees of differentiation. *I^2^* estimates showed no heterogeneity among the studies (*I^2^* = 71%, χ^2^ = 17.25, *P*=0.004), and the random-effects model was used for this meta-analysis. It was indicated that the expression level of OCT-4 protein in poorly differentiated CC is higher than in well-differentiated and moderately differentiated CC, however, the difference is not statistically significant (OR = 0.40, 95% CI: 0.09–1.71, *P*=0.22, Figure 6).

##### Differential expression of OCT-4 in CC with lymph node metastases: positive group vs. negative group

**Figure 7:**
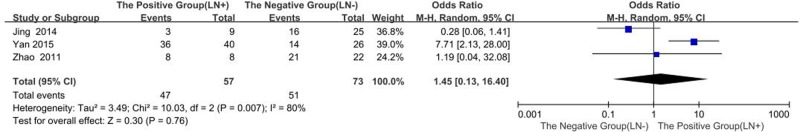
Meta-analysis of expression of OCT-4 protein in CC tissues with positive group vs. negative group lymph node metastases

A total of three studies reporting OCT-4 overexpression in positive and negative lymph node metastases groups of CC tissues. *I^2^* estimate showed heterogeneity among the studies (*I^2^* = 80%, χ^2^ = 10.03, *P*=0.007), and the random-effects model was used for this meta-analysis. The results showed that there is no significant difference in OCT-4 protein expression in patients with lymph node metastases compared with those without (OR = 1.45, 95% CI: 0.13–16.40, *P*=0.76, Figure 7). It is speculated that the expression of OCT-4 protein in CC is not significantly associated with the occurrence of lymph node metastasis.

##### Differential expression of OCT-4 in different stages of CC: FIGO I vs. FIGO II

**Figure 8:**
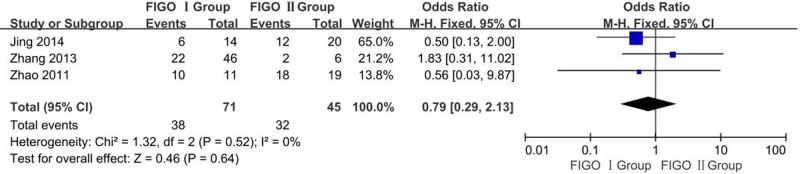
Meta analysis of differences in expression of OCT-4 protein in different stages of CC

A total of three studies reporting OCT-4 overexpression in FIGO I and FIGO II of CC tissues. *I^2^* estimate showed heterogeneity among the studies (*I^2^* = 0%, χ^2^ = 1.32, *P*=0.52), and the fixed-effects model was used for this meta-analysis. There is no significant difference in the expression of OCT-4 protein among different FIGO stages of CC (OR = 0.79, 95% CI: 0.29–2.13, *P*=0.64, Figure 8).

##### Differential expression of OCT-4 in CC squamous cell carcinoma vs. adenocarcinoma

**Figure 9:**
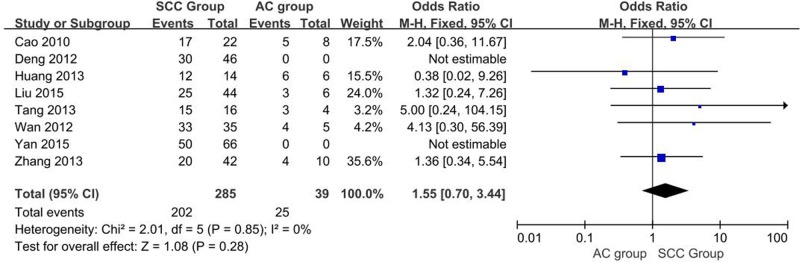
Meta analysis of differences in expression of OCT-4 protein in CC tissues with different pathological types

A total of eight studies reporting OCT-4 overexpression in different pathological types of CC tissues. *I^2^* estimate revealed significant heterogeneity among the studies (*I^2^* = 0%, χ^2^ = 2.01, *P*=0.85), and the fixed-effects model was used for this meta-analysis. There is no significant difference in the expression of OCT-4 protein between squamous cell carcinoma (SCC) and adenocarcinoma; (AC) (OR = 1.55, 95% CI: 0.70–3.44, *P*=0.28, Figure 9).

### Publication bias

No publication bias in studies including in the literature related to lymphatic metastasis, differentiation and FIGO staging (*P*>0.05), whereas studies on the pathological type had publication bias (*P*<0.05).

## Discussion

CC is a common gynecological tumor that threatens women’s health. CSCs are characterized by self-renewal, multi-directional differentiation, radiotherapy and chemotherapy resistance, high tumorigenicity and so on, and may affect the invasion, metastasis, and recurrence of CC [30–32].

The discovery of CSCs provides new possibilities for diagnosing and treating CC. Scholars have conducted extensive research on CSCs related to CC. In 2012, Lopez et al. [33] found cancer-initiating cell subsets with self-renewal ability in the CC cell lines HeLa, SiHa, Ca Ski and C-4I, which can express the characteristic markers of stem cells, epithelial mesenchymal transition (EMT) and radioresistance. Liu et al. [34] explored the functional relationship between endogenous nuclear protein SOX2 expression and cervical cancer CSCs, and revealed that isolating CSCs from CC somatic tumors is feasible. Ortiz-Sánchez et al. [35] confirmed that the expression of CK-17, p63+, AII+, CD49f+ in CC stem cells and activity of aldehyde dehydrogenase (ALDHbright) are high. Tyagi et al. [36] confirmed that CSCs isolated from CC have self-renewal ability and could express the markers OCT-4, Sox2, Nanog, Lrig1 and CD133. Javed et al. [37] found that CD133 positive in CC stem cells, and increased expression in recurrent CC, increased CD133-positive tumor cell formation, and up-regulated EMT-related markers.

OCT-4 expression is closely related to the occurrence of malignant tumors and affects their rapid development, high metastasis rate, and poor prognosis, thus suggesting that the protein can predict the occurrence and development of tumors and be regarded as a prognosis indicator [38,39]. OCT-4 plays a dual role in the non-differentiated cell stages. In addition to maintaining cells in the non-differentiated stage, OCT-4 can differentiate cells into other types. While knockout of OCT-4 results in the disappearance of, cell differentiation and stem cell characteristics, the transcription factor retains its self-renewal and infinite amplification abilities, thereby resulting in the occurrence of tumors [40,41].

CC is related to HPV infection, especially high-risk HPV16 and HPV18 [42]. Liu et al. [43] predicted that HPV infection may accelerate the up-regulation of OCT-4 expression to trigger CC, and suggested that OCT-4 may present a new type of targeted therapeutic approach for CC. His team [44] further proved that HPV16 is the key factor triggering the occurrence and development of CC. The expression of OCT-4 is up-regulated in CC cells and may be activated by HPV16 infection; in fact, its expression level in HPV16-infected CC cells is higher than that in non-infected patients [45].

Kim et al. [46] found that OCT-4 expression in CC cells is higher than that in the normal cervix, and that OCT-4 overexpression is associated with space invasion of CC lymph vessels. Compared with the low expression of OCT-4, progression-free survival (PFS) and overall survival (OS) are worse in 5 years in the overexpression group, thereby suggesting that OCT-4 is an independent risk factor of CC and that patients with OCT-4 overexpression suffer from poor prognoses. OCT-4 increases progressively from the normal cervix to CIN and then to CC. Shen et al. [47] showed that OCT-4 overexpression in the radiation-resistant group is considerably higher than that in the radiation-sensitive group and that PFS in the OCT-4 overexpression group decreases. Thus, OCT-4 protein could predict radiation resistance in locally advanced cervical SCC (LACSCC) patients and poor survival rates. Considering these findings, OCT-4 is an independent prognostic factor of CC, and may represent a potential target for CC treatment [48].

Many miRNAs, such as miR-145, miR-302, miR-125b, miR-430, miR-200, miR-335, are associated with OCT genes [49]. OCT-4 induces miR-125b expression in certain means, thereby inhibiting BAK1 expression and, ultimately, the apoptosis of CC cells. Multiple miRNAs are involved in the regulation of tumor growth, metastasis of tumor, and chemoradiotherapy sensitivity by interacting with OCT-4 [50]. Yan et al. [42] confirmed that miR-145 could improve the sensitivity of CC radiation by inhibiting OCT-4 expression; this finding suggesting that cyclin D1 positively regulates OCT-4 and mediates the function of miR-145.

In the present study, the clinicopathological features of OCT-4 and CC were collected for statistical analysis and results indicated that OCT-4 expression is higher in CC than in CIN and normal tissues. This result is consistent with the findings of Huang et al. [21,23,24], who suggested that OCT-4 overexpression increases the risk of CC. Among different clinical and pathological features, OCT-4 expression shows different results. Yan et al. [27] believed that OCT-4 expression has significant meaning for CC lymphatic metastasis, and increases the risk of poor prognosis. Zhao et al. [21] and Ji et al. [29] reported that OCT-4 expression is not involved in CC lymphatic metastasis. Deng et al. [21–23,25,27,28] found that OCT-4 expression is significantly related to histological differentiation; Jing et al. [24,26,29], however, denied this argument. Meta-analysis showed that the lower differentiation of CC, the higher the expression of OCT-4, which indicates that OCT-4 overexpression is closely related to the occurrence and histological differentiation of CC. By contrast, OCT-4 expression is not significantly related to lymphatic metastasis, FIGO stage, and pathological type.

The meta-analysis features publication bias as follows. (i) Although Korean researcher Kim [41] reported that OCT-4 is expressed in CC, the relevant data could not be extracted and included. The study area was in China, and a simple model of population was applied; thus, extrapolation of findings is limited. (ii) Both Chinese and English literature were included in this work, but the gray literature was insufficient. This limitation may result in missed negative results and publication bias. (iii) The included studies were of moderate quality, which may obscure the unknown defects of the design. (iv) The inclusion criteria of certain studies were minimal, and the conclusions must be further verified. (v) The studies all applied the IHC method to detect OCT-4 expression, but the antibody manufacturers, dilution concentration, and judgment criteria were not identical, which may affect the results.

In conclusion, OCT-4 expression is higher in CC than in CIN and normal tissues. Such a result is related to histological differentiation and is not significantly correlated with lymphatic metastasis, FIGO stage, and pathological type, which indicates that OCT-4 may play important role in the occurrence and progression of CC. The findings also suggest OCT-4 may serve as a prognostic indicator of CC and potential target of stem cell therapy. However, rigorous, less biased, and high-quality randomized controlled studies based on NOS standards are needed for further confirmation of the present findings.

## Abbreviations

CC: cervical cancer
CI: confidence interval
CIN: cervical intraepithelial neoplasia
CSC: cancer stem cell
EMT: epithelial mesenchymal transition
ESC: embryonic stem cell
IHC: immunohistochemistry
NOS: Newcastle–Ottawa Quality Assessment Scale
OCT-4: octamer binding transcription factor 4
OR: odds ratio
PFS: progression-free survival
SCC: squamous cell carcinoma
